# The morphophonological dimensions of Spanish gender marking: NP processing in Spanish bilinguals

**DOI:** 10.3389/fnhum.2024.1442339

**Published:** 2024-12-13

**Authors:** Ana T. Pérez-Leroux, Laura Colantoni, Danielle Thomas, Crystal H. Y. Chen

**Affiliations:** ^1^Experimental Microvariation Lab, University of Toronto, Toronto, ON, Canada; ^2^Department of Spanish and Portuguese, University of Toronto, Toronto, ON, Canada; ^3^Department of Linguistics, University of Toronto, Toronto, ON, Canada

**Keywords:** Spanish gender, heritage speakers, NP processing, eye-tracking, bilingual processing, domain interactions, vowel centralization, bilingual effects

## Abstract

The processing literature provides some evidence that heritage Spanish speakers process gender like monolinguals, since gender-marking in definite articles facilitates their lexical access to nouns, albeit these effects may be reduced relative to speakers who learned the language as majority language. However, previous studies rely on slowed-down speech, which leaves open the question of how processing occurs under normal conditions. Using naturalistic speech, our study tests bilingual processing of gender in determiners, and in word-final gender vowels. Participants were 17 adult heritage speakers of Spanish (HSSs) and 21 adult Spanish-speaking immigrants (ASIs). We presented these bilinguals with questions containing either a definite article or an unmarked possessive (*¿Dónde está la/mi pala?* ‘Where is the/my shovel?’) in a three-object display. Gaze fixations were recorded during determiner, noun and post speech processing. Nouns were controlled for gender, morphological transparency, gender alternation, and animacy. Individually, heritage speakers tend to fall within the performance range of adult immigrants, but statistical analyses show that ASIs have more fixations to targets for definite articles compared to HSSs. For HSSs the advantage of gender-marking appears later, during noun processing. In contexts where the noun-final vowels were the only cue to lexical selection, HSS had less looks to targets with alternating nouns, and with feminine nouns. When presented with natural speech, despite the great overlap between adult immigrant and heritage speakers, there are quantitative differences in how HSS process gender both for syntactic agreement (gender in articles) and noun morphophonology.

## 1 Introduction

Spanish gender is a category that partitions nouns into morphological classes and can express a biological sex in animate referents (*el gato y la gata* ‘the male and the female cat’), or lexical differences (*el palo* ‘stick’ vs. *la pala* ‘shovel’). For a large portion of the nominal lexicon in Spanish, gender is expressed by a word-final vocalic word marker (unstressed vowels -o and -a). In sentence processing, gender-marked articles have been shown to support lexical processing of noun phrases (NPs) (Lew-Williams and Fernald, 2007a,b). To what extent is this ability available in heritage bilinguals? The current study aims to contribute to our understanding of the mental organization of grammar in heritage speakers (Polinsky and Scontras, 2020), by examining potential differences in how two groups of adult Spanish bilinguals use gender information on a determiner and in word final vowels. While previous work has explored the processing effects of gender in the determiner, no studies have directly examined how bilinguals process gender when the word-final marker is the only cue to gender. Because of the potential phonetic variability in unstressed word-final vowels in bilingual speech perception and production, we are also particularly interested in bilingual processing under natural speech conditions.

Spanish gender is considered transparent and predictable, with over 60% of nouns ending in the transparent word final markers /o a/ (masculine and feminine, respectively), with a smaller percentage of /e/−ending nouns (13%) being predominantly masculine (Clegg, 2011). Research indicates that children rely on phonological information to build grammatical categories (Culbertson et al., 2019). More specifically, Spanish-speaking young children rely heavily on phonological cues to assign gender to new words (Perez Pereira, 1991). At the same time, it is known that phonetic variability in the input to a given form can delay acquisition of a marker (e.g., Miller and Schmitt, 2010). What happens in heritage populations, when the phonetic input to gender is not reliable due to language contact? This is the case of Spanish heritage speakers who grew up in a community in contact with English. Spanish has a five-vowel system that remains stable in stressed and unstressed positions (Navarro Tomás, 1970; Delattre, 1965; Hualde, 2014). In contrast, the large vowel inventory of English (Labov et al., 2006; Ladefoged, 2001) is drastically reduced to one or two phonemes in unstressed position (Rogers, 2000). Unsurprisingly, unstressed vowels merge and centralize in heritage Spanish-English speaking children (Gildersleeve-Neumann et al., 2009; Menke, 2010; Lease, 2022) and adults from various regions in the US (New Mexico, Willis, 2005; North Carolina, Boomershine, 2012; Midwest, Ronquest, 2012, 2013; Shea, 2019; Florida, Alvord and Rogers, 2014; El Paso, Colantoni et al., 2020). Mazzaro et al. (2016) find that difficulties in unstressed vowel perception persist into adulthood.

Linguistic studies indicate that grammatical gender develops differently in heritage bilingual children, Eichler et al. whose lexical and syntactic gender errors persist through the school years (Eichler et al., 2013; Montrul and Potowski, 2007; Morgan et al., 2013; Martínez-Nieto and Restrepo, 2023; Pérez-Leroux et al., 2023). Until recently, the question of whether phonological factors (vowel centralization) and gender accuracy are associated had not been explored. Studies on narrative data by Colantoni et al. (2020) and Pérez-Leroux et al. (2023) included an acoustic analysis of unstressed final /a e o/. Their results show great variation in individual gender grammars in heritage adults and children, and generally intact gender systems in adult sequential bilinguals. In terms of the realization of word-final vowels, their data show substantive phonetic overlap in bilingual children and adults with early bilingual exposure. However, these two studies do not find a correlation between measures of vowel overlap and accuracy in gender agreement and assignment. This may be attributed in part to the quality of the data, which allowed for the acoustic analysis of a subset of the data. Moreover, these studies correlated vowel production with gender accuracy, but perception and comprehension data were not available.

Grosjean et al.’s (1994) and Guillelmon and Grosjean’s (2001) pioneer findings demonstrated that speakers of gender-marked languages use prenominal gender marking to process the noun. This observation has been validated across languages (Baron et al., 2022). In Spanish, various Visual World Processing (VWP) eye-tracking studies have examined whether listeners can use the gender information on the definite article (*el* vs. *la*) to predict an upcoming noun. Lew-Williams and Fernald (2007a, 2007b) presented monolingual children and adults with NPs containing gender-marked articles and two objects on a screen. Listeners’ gazes fixated faster to the target noun when the two nouns were of different gender than when the two nouns were of the same gender, suggesting that they could use the gender of the determiner to facilitate referent identification. Other studies have confirmed this result, and further explored how bilingualism affects the listener’s ability to use grammatical gender predictively in processing. Studies included factors such as age of exposure and type of bilingualism (e.g., heritage, late L2 English, late L2 Spanish, Lew-Williams and Fernald, 2009, 2010; Fuchs, 2022), type of language proficiency and/or abilities (e.g., productive vocabulary, Dussias et al., 2013), current language use (Baron et al., 2022), linguistic identity of L1 (e.g., Italian or English, Dussias et al., 2013), and a combination of these factors (see Table 1 for a summary).

**Table 1:** Gaze studies of processing of Spanish gender.

Publication	Populations	Trials design	Auditory stimuli	Main findings
Lew-Williams and Fernald (2007a)	Monolinguals in US: Children (2;8–3;6) (*n* = 26) Adult (*n* = 26)	Definite determiner 2 objects (same/diff gender trials)	Unspliced audio; article duration: 268–299 ms. (Slow)	Child and Adult monolinguals recently arrived in US: Effect of trial; adults faster than children
Lew-Williams and Fernald (2007b)	Controls are same participants in 2007a; L2 adults (*n* = 33)	Same as 2007a	Same as 2007a	Late L2 adults have no differences in trial type
Lew-Williams and Fernald (2009)	US Adults: Heritage bilinguals (“L1 adults”); L2: late learners	2 objects (same/diff gender trials) Exp. 1 and 2: Novel nouns with Def and Indef; Exp 3 human	Unspliced audio; article duration 268–299 ms. (Slow)	Effect of same/different For familiar nouns: Heritage = L2 For novel nouns: Heritage > L2
Lew-Williams and Fernald (2010) (Comparing 2007 and 2009)	Heritage adults L2 adults (*n* = 26)	Exp 1 (familiar nouns): Same as 2007a Exp 2 (Def) and 3 (Indef) (novel nouns): same as 2009	Same as 2007a	Familiar nouns: Children and Heritage show effect of trial; L2, no effect Novel Ns + Def, both adult groups show effect; with Indef, only Heritage adults show effect.
Gruter et al. (2012) (re: L-W and F. 2007a/2009)	Late immigrants to US (‘L1’) (*n* = 19), Late L2 Spanish, near-natives (*n* = 19)	2 objects (same/diff gender trials) Exp 1: Def + familiar N Exp 2: Indef + novel N	Same as 2007a	For familiar nouns, effect in both groups, with smaller effect in L2. For novel nouns: comparable effects.
Dussias et al. (2013)	Adults in Spain: Monolingual (*n* = 16) English L1: Low and High L2 Spanish (*n* = 18); Italian L1, Low Spanish (n = 15)	2 objects (same/diff gender trials) Controlled position of noun in sentence	Unspliced audio; article duration edited: 147 ± 3 ms.	No effect of position; Monolinguals and High L2 have effect of trial; Low L2 group show no effect; for feminine, effect shown in Italian L1 only.
Morales et al. (2016)	Adult learners in Spain: Italian proficient L2 speakers (*n* = 32)	2 object arrays with same gender Congruent/incongruent gender relative to Italian	Unspliced audio; article duration edited: 147–200 ms + 50 ms gap (Slow)	Faster to target in trials with lexemes that are gender-congruent to Italian translation equivalents.
Valdés Kroff et al. (2017) (Exps., 1a and1b)	Monolinguals (*n* = 24); immigrants before adulthood (*n* = 25)	2 objects (same/diff gender trials) (Spanish only, non-codeswitched study)	Spliced audio; article duration: 200 ms + 50 ms silence (Slow)	Monolinguals: effect of trial, no differences for gender. Bilinguals have effects for feminine only.
Halberstadt et al. (2018)	Adults Monolinguals in Spain (*n* = 23); Adult L2 Advanced in US (*n* = 18)	2 objects (same/diff gender trials); items controlled for transparency and cognate status	Spliced audio; article duration: 147 ± 3 ms	Monolinguals: effect of trial; no effect of cognate status or transparency. L2: Trial effects only for transparent items.
Fuchs (2022)	Adult US Late immigrants (*n* = 10); Heritage (*n* = 20)	2 objects (same/diff gender trials)	Spliced audio; article duration: 280 ms (Slow)	Effect of trial in both groups; magnitude of effect is greater for late immigrant controls.
Baron et al. (2022)	Child heritage bilinguals in US (5;6–8;6) (*n* = 32) Split by levels of BESA Spanish use	4 objects array (all same or target is different); Measured anticipation (effect before noun) and facilitation (after noun)	Spliced audio; Masc article: 365 ms Fem article: 300 ms (Slow)	No anticipation effect; facilitation only in children with “more Spanish use,” for feminine nouns, effect only in Italian.

Overall, child and adult Spanish speakers born and educated in a Spanish-speaking country show differential gaze behaviors for same vs. different trials, regardless of their current residence. While transparent nouns are generally more accurate but slower to retrieve in lexical decision tasks than opaque/gender ambiguous nouns (Sá-Leite and Lago, 2024), eye-tracking studies of Spanish monolinguals have neither determined whether noun transparency mediates article facilitation effects (Caffarra et al., 2017; Halberstadt et al., 2018), nor directly focused on how the vowel is processed when it is essential for lexical decision (in alternating nouns) or redundant with the retrieval of the noun lexeme (in non-alternating nouns).When comparing groups, studies have suggested that adult native speakers (monolinguals and bilinguals of late L2 English) process trials faster than monolingual children, but the magnitude and directionality of the difference between the same vs. different trials is similar (earlier or faster looks to target for “different” trials). In contrast, results for late learners of Spanish (i.e., L2 speakers) vary. Studies not specifically examining L2 Spanish proficiency do not show differential gaze behaviors for “same vs. different” trials (Lew-Williams and Fernald, 2007b, 2009). However, in a series of studies, Lew-Williams and Fernald (2009, 2010) showed that despite not showing differential gaze behaviors on the processing of familiar Spanish nouns, L1 English-L2 Spanish speakers evidenced differential gaze behaviors for trial-type when the targets are human referents. Other studies have measured relative Spanish proficiency and shown some effects of predictive processing in late L2 Spanish speakers. In Dussias et al. (2013), L1 Italian-L2 Spanish speakers with low proficiency exhibit differential gaze behavior for feminine objects only, while their English counterparts with low Spanish proficiency do not exhibit evidence of predictive processing. There is evidence of sensitivity for “same vs. different gender” trials for L2 speakers with higher levels of proficiency, often with smaller magnitude of differences than what was found for native Spanish-speaking adults (Gruter et al., 2012; Dussias et al., 2013). Recent evidence further indicates that this predictive effect only occurs for target objects with transparent noun morphology (o/a endings) (Halberstadt et al., 2018).

A few studies focus on speakers with early exposure to bilingualism. For this population, results are also mediated by various language-internal and language-external factors. Lew-Williams and Fernald (2009, 2010) compare L1 and L2 adults living in the US. The L1 Spanish speakers in these studies are described as US residents exposed to Spanish since birth, and likely best characterized as heritage Spanish speakers. These speakers appear to exhibit differential gaze behavior patterns on same vs. different trials, which resembles the findings for the adult monolinguals in Lew-Williams and Fernald (2007a, 2007b). Fuchs (2022) replicated these results by showing that adult heritage speakers of Spanish in the US exhibit differential gaze behaviors for same vs. different trials, but the magnitude of the difference is smaller than those of native speakers of Spanish who arrived late to the US. Other work indicates differential effects depending on the gender of the trial. Valdés Kroff et al. (2017) showed that adult native Spanish speakers who had first been exposed to English at around 9 years of age through immigration to the US exhibited differential gaze patterns for feminine nouns only.^1^ This contrasts with the performance of adult monolinguals in the same study who exhibited predictive effects for masculine and feminine objects equally. Finally, Baron et al. (2022) examined gender processing in heritage bilingual children in Texas (5;6-8;6). These authors distinguish between anticipatory gaze effects, occurring before the auditory onset of the noun, and facilitative gaze effects, those occurring from the onset of the noun until 1,150 ms after the noun onset. No child exhibited anticipatory gaze behaviors, but a group difference emerged for “facilitative” gaze behaviors. Those with “more current Spanish use” exhibited facilitative gaze effects for feminine nouns only, while those with “less current Spanish use” did not exhibit any predictive gaze effects.

In general, listeners build quick, grammatically accurate representations, making effective use of available syntactic/semantic knowledge, although there might be differences between populations of speakers (mature vs. learners; monolingual vs. bilinguals; see Phillips and Ehrenhofer, 2015). At the same time, studies of online comprehension are highly sensitive to lexical and methodological effects, which need to be considered when we examine how effective various types of speakers are at predicting upcoming material in sentence comprehension. In eye-tracking studies, beyond image saliency, word frequency is a key factor that influences processing. Frequency affects the earliest moments of lexical access, with listeners’ gazes fixating earlier for high frequency nouns (Dahan et al., 2001). Gender is both a syntactic property (agreement unifies nominal constituents) and a lexical property. Many lexical characteristics of gender have shown to be potentially relevant dimensions of performance, at least in bilinguals. In addition to lexical frequency, other factors also impact processing of grammatical gender in a VWP task. Let us discuss some of the main factors.

### 1.1 Gender markedness

Markedness may yield an asymmetry, where speakers use gender information differentially in processing tasks. In Dutch, studies examining predictive effects of prenominal gender show asymmetrical effects, but mixed results (common advantage for Dutch adults in Loerts et al. (2013), and neuter advantage for Dutch adults and children in Brouwer et al., 2017). In Spanish, where the masculine is considered the unmarked form, facilitation occurs only for the marked (feminine) form in some studies and populations (Valdés Kroff et al., 2017; Baron et al., 2022). ERP testing supports a feminine advantage in the detection of grammatical violations in Spanish Det-N phrases by adult monolinguals (Beatty-Martínez, 2019).

### 1.2 Transparency

Some nouns have transparent gender morphology, where *-o* ending is masculine and *-a* ending is feminine; words with the opposite pattern are exceptional, presenting learning challenges. Other word endings are opaque (−*e* is frequently masculine, but the association is more ambiguous; many consonantal endings have no strong association, although many derivational morphemes do; Clegg, 2011). Non-transparent words show higher error rates in bilinguals (Martínez-Nieto and Restrepo, 2023). Halberstadt et al. (2018) is one VWP eye-tracking study that controls for transparency, finding that late L2 bilinguals only show an advantage of gender when processing transparent nouns.

### 1.3 Speech

In nearly all previous VWP studies examining Spanish gender, the type of auditory stimuli consisted of spliced or slow speech, argued to be necessary to avoid effects of speech co-articulation ^2^ (Fuchs, 2022). To facilitate time-course analysis, most studies have manipulated the audio so that the onset of the determiner and/or noun fall at the same point in time across trials. Therefore, for the studies that have determiner durations more typical of normal speech (e.g., Dussias et al., 2013), the audio is time-normalized to facilitate the time-course analysis. The longer latencies and manipulated stimuli facilitate time course analysis and give enough time for eye movements to occur.

From our perspective, use of manipulated speech raises the question of whether listeners use information predictively on the Spanish determiner when listening to naturalistic speech. Furthermore, we must consider potential issues with phonetic cues. Colantoni et al. (2020) show that in narratives, 40% of nouns are not redundantly marked for gender, i.e., they are not preceded by an informative determiner (e.g., definite or indefinite), and the only information for gender is in the final vowel of the noun. If we consider the centralization of unstressed (final) vowels in Spanish-English bilinguals, we are forced to contemplate the possibility that canonically marked alternants might be phonologically merged, i.e., /ˈpala/ and /ˈpalo/ (‘shovel’ vs. ‘stick’) can become /ˈpalə/. We propose to examine whether vowel alternation plays a role in bilinguals’ use of gender in sentence processing. Our study departs from previous Spanish studies (i) in the use of unmanipulated audio stimuli; (ii) in the manipulation of the type of determiner: gender-informative (i.e., definite determiner *el/la*) vs. gender uninformative (possessive *mi* ‘my’, both F/M), as in work by Grosjean et al. cited above; and (iii) in exploring the specific role of the final vowel in the processing of noun phrases where the contrast between vowels is the only exponent of gender, i.e., in the context of determiners that are unmarked for gender, such as the possessive. Although markedness in general may give an advantage in processing, at least for L2 speakers, one might anticipate that some heritage speakers (those with undifferentiated unstressed vowel inventories) might struggle in these contexts.

Thus, our study has three goals. The first goal is to determine if Spanish speakers can use gender information on a determiner to predict the upcoming noun referent when presented with stimuli at normal speech rates. The second goal is to examine whether this is also true of heritage speakers, relative to those who grew up in a Spanish-speaking context. Our third goal is to explore how gender in the Spanish NP is processed in transparent nouns when the prenominal determiner is unmarked for gender.

## 2 Materials and methods

### 2.1 Participants

Thirty-eight Spanish-English bilinguals living in Canada participated in this study. The first group (*n* = 17) included adult Heritage Speakers of Spanish (HSS), who were either born in Canada/US or moved to Canada during primary school (i.e., before 11 years old) and whose home language was predominantly Spanish. A second group (*n* = 21) consisted of adult Spanish-speaking Immigrants (ASI), who were born and raised in Spanish-speaking countries and moved to an English-speaking country during secondary school or later. The HSS participants (mean age: 23.7) in this study were recruited in social networks on the basis of their oral communicative abilities in Spanish. All communication during recruitment and participation was in Spanish, and all participants self-rated as having a “native” or “superior-advanced” global competency in both Spanish and English. The ASI participants (mean age: 26.7) were born and did most or part of their high school education in a Spanish-speaking country. Most of these participants had moved after 18 years of age. Two participants had moved to Canada during secondary school (~15 years old).^3^ Most participants had reported learning English in primary and/or secondary school, but none had English-speaking family members or immersion experiences in an English language context as children. ASIs self-rated as having “native” global competency in Spanish, and “intermediate,” “advanced,” or “superior” global competency in English.

Participants were recruited in Toronto, a heavily multilingual city, where approximately 45% of residents speak one or more non-official languages at home. While Spanish is among the five topmost common languages (other than English), and has grown in the last two decades, only a small percentage of the population has Spanish as the mother tongue (2.8%) with many stating they use their home language regularly (1.7%) (City of Toronto, 2021). Our participants belong to the same language communities, which are both sparse and scattered across the city. Parents raising children with Spanish at home report their families having modest access to language resources and to other speakers (Pérez-Leroux et al., 2011).

### 2.2 Tasks

#### 2.2.1 Experimental design

We designed a study to answer the following research questions:

- Can HSSs use gender on the determiner to facilitate processing of an upcoming noun when faced with naturalistic input and in more complex settings?
- How do HSSs compare to speakers that immigrated after adolescence (ASIs)?
- Can HSSs use noun-final vowels /−o/ and /−a/ to disambiguate objects, in the absence of a gender cue on the possessive determiner?

Previous gaze studies of Spanish gender followed the common approach of maintaining the type of determiner constant and varying the gender of distractors. We maintained a fixed visual array, while counterbalancing the type of determiner (gender-informative definite *la pala* ‘the shovel’, vs. ambiguous possessives *mi pala* ‘my shovel’) across participants.

Our trials also increased task complexity by using a 3-image array: a Target noun, its phonological and gender alternant or Competitor, and a Distractor, which was phonologically unrelated to the Target and Competitor objects. Our study controlled for transparency in nominal morphology (i.e., whether the noun contains the expected gender vowel /a o/ or ends with a final consonant or /e/).

The nouns and objects in our trials were selected to present a diversity of entities and array types. Target nouns were either morphologically transparent or opaque. Competitor images depicted (a) the gender alternate of a transparent alternating noun, i.e., differentiated only by the final vowel (*n* = 16, *palo/pala*, ‘stick/shovel’), or (b) for non-alternating nouns, i.e., the image of a noun with opposite gender and similar phonological onset (*sombrero/sombrilla*, ‘hat/umbrella’) to the target. Non-alternating target nouns could be either transparent or opaque. In addition to controlling for morphology and grammatical gender, we also considered other variables such as animacy, which may influence gender processing (Lew-Williams and Fernald, 2009; Halberstadt et al., 2018), and the gender of the distractor object. The study included 48 experimental trials. Table 2 summarizes the types of nouns included.

**Table 2:** Study design: lexical and morphological types included.

Variable	Conditions	Tokens	Example
Target Gender	Masculine Feminine	*t =* 24 *t =* 24	palo*, stick* casa*, house*
Noun transparency	Transparent Opaque	*t =* 31 *t =* 17	palo (m) / casa (f)^a^ *stick / house* sobre (m) / nube (f) *envelope / cloud*
Animacy	Inanimate Human Animal, alternating Animal, epicene	*t =* 24 *t =* 11 *t =* 4 *t =* 9	pal**o**, *stick* maestr**o**, *teacher* os**o**, *bear* sap**o**, *toad*
Gender alternation	Minimal pairs Other phonological	*t =* 16 *t =* 32	**pal**o / **pal**a *stick / shovel* **sombr**ero / **sombr**illa *hat / beach umbrella*
Distractor Gender	Same as target Different	*t =* 24 *t =* 24	**sombrero-**sombrilla-**martillo** *house-car-guitar* **palo**-pala-cuchara *stick-shovel-spoon*

a One transparent trial was converted to opaque during piloting as the result of lexical preferences identified.

**Figure 1:**
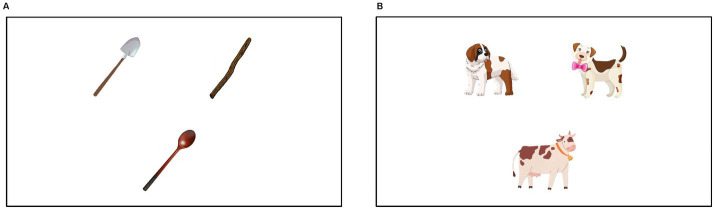
Examples of inanimate **(A)** and animate trials **(B)**.

We designed color drawings for most of the images in our study but also included images from open-source repositories (e.g., the Snodgrass-Vanderwart pictures, see Sanfeliu and Fernandez, 1996). To avoid gaze response biases detected in previous studies (Huettig and McQueen, 2007; Hintz et al., 2020), the image arrays contained objects as comparable as possible in shape, size, and colouring and other visual characteristics. The three image sets contained objects in the same animacy class (inanimates, animals, and humans),^4^ and we endeavored to select visually comparable sets, including types of animals, and inanimate objects within close semantic fields (tools, etc.). Three images were situated on all trials in an upside-down triangle formation, with the centre of each image being equidistant from the centre point of the 1,920×1,080 screen. Each image centerpoint was located at 200 pixels from centerpoint (bottom image) and 250 pixels from centerpoint of screen (two top images). Gaze points were captured in standard sized areas of interest: rectangles 500 pixels high and 480 pixels wide, with a minimum of 50 pixels of space between closest boundaries of the three AOIs. These AOIs capture all fixations directly on image, plus an average of 100 pixels in surrounding space (see Figure 1).

The location of the Target, Competitor, and Distractor images was semi-randomized, so that each object type (T, C or D) appeared in all positions (top L, top R, bottom) for each condition. Appendix 1 provides a list of the practice and experimental trials tested in this study.

### 2.3 Auditory stimuli

The current study examines how Spanish speakers process speech with natural timing, i.e., neither spliced nor slowed down. Our stimuli consisted of interrogative sentences in Spanish constructed using a carrier phrase (*Dónde está (…)?* ‘Where is (…)?’), followed by a determiner + noun. A native female speaker of Mexican Spanish was recorded reading each target sentence three times, using casual speech. A phonetician selected the best sentence prompt for each trial to use in the experiment. On average, the speech rate of our stimuli was 6.6 syllables per second, with a range from 5.7 to 7.7 syllables per second. This falls within expected ranges for the Mexican variety (Santiago and Mairano, 2022). The nouns ranged from 1 to 4 syllables. Subsequently, we normalized prompts for intensity (70 dB) and used Praat (Boersma and Weenink, 2021) to measure sentential duration and F1/F2 for vocalic nouns endings /a e o/ to ensure that the stimuli expressed the expected acoustic vowel quality.

Consistent with naturalistic speech, the duration of each sentential component (carrier phrase, determiner, and noun) varied from trial to trial, and the acoustic quality of word-final unstressed vowels /a e o/ fell within the expected range of F1 and F2 values for Spanish. Table 3 summarizes the ranges of the sentential components for the auditory stimuli in milliseconds.

**Table 3:** Durational ranges for auditory stimuli.

Sentential component	Constituent	Range in ms.	Mean in ms	SDs
Full sentences, n = 96	*¿Dónde está [det-N]?*	838–1,546	1107.9	123.0
Masculine definite *t* = 24	*el*	70–166	103.3	21.3
Feminine definite *t* = 24	*la*	101–153	124.9	14.0
Possessive, *t* = 48	*mi*	95–202	143.2	19.1
Noun, *t* = 96		212–762	488.4	106.9

Gaze analyses were planned for pre-defined temporal windows (see Morales et al., 2016). The first window, determiner processing, starts at the onset of the determiner and lasts for the duration of the determiner plus 200 ms, the average time which has been shown to be required to launch a saccade upon hearing an auditory stimulus (Fischer, 1999, *cf.* Dussias et al., 2013). The second window, noun processing, starts with the onset of the noun plus 200 ms and lasts for the duration of the noun plus 200 ms. The third window, post speech, starts at the offset of the noun +200 ms and lasts for 400 ms. Given the nature of our stimuli, these windows have variable durations.

### 2.4 Procedure

After signing their consent to participate in the study and to be audio-recorded, participants completed a picture-naming task. The purpose of this task was to ensure that each participant had knowledge of the Spanish lexical item targeted in the eye-tracking study and associated them with the object images employed. Participants were asked to name an object on the screen, and then a recording supplied the name of the object with an indefinite article. This phrase was delivered by an animated small blue bird on the screen. The participant was then asked to repeat the phrase heard for confirmation. Participant responses were manually recorded during the session and then later verified from the audio recording by a second rater.

Following the picture-naming task, participants engaged in a short language background interview, and then began the eye-tracking task. Gaze was recorded on a Tobii ProFusion eye-tracker with a sampling frequency of 120 Hz. The participants sat 60–70 cm from a color monitor and completed a 9-point calibration and validation procedure. Participants would proceed onto the eye-tracking task if the average error on the calibration and validation modelling was <0.5 degrees.

Participants saw an instruction screen, received verbal instructions, and then completed six practice trials where they became familiar with the task. For each trial, participants saw the little blue bird in the middle of the screen for 800 ms while a whistling noise played to centre their attention. Following this fixation, the 3-image array appeared on the screen for 2 s of preview time. After 2 s, the audio stimulus played, *¿Dónde está…?* (‘Where is…?’).

**Figure 2:**
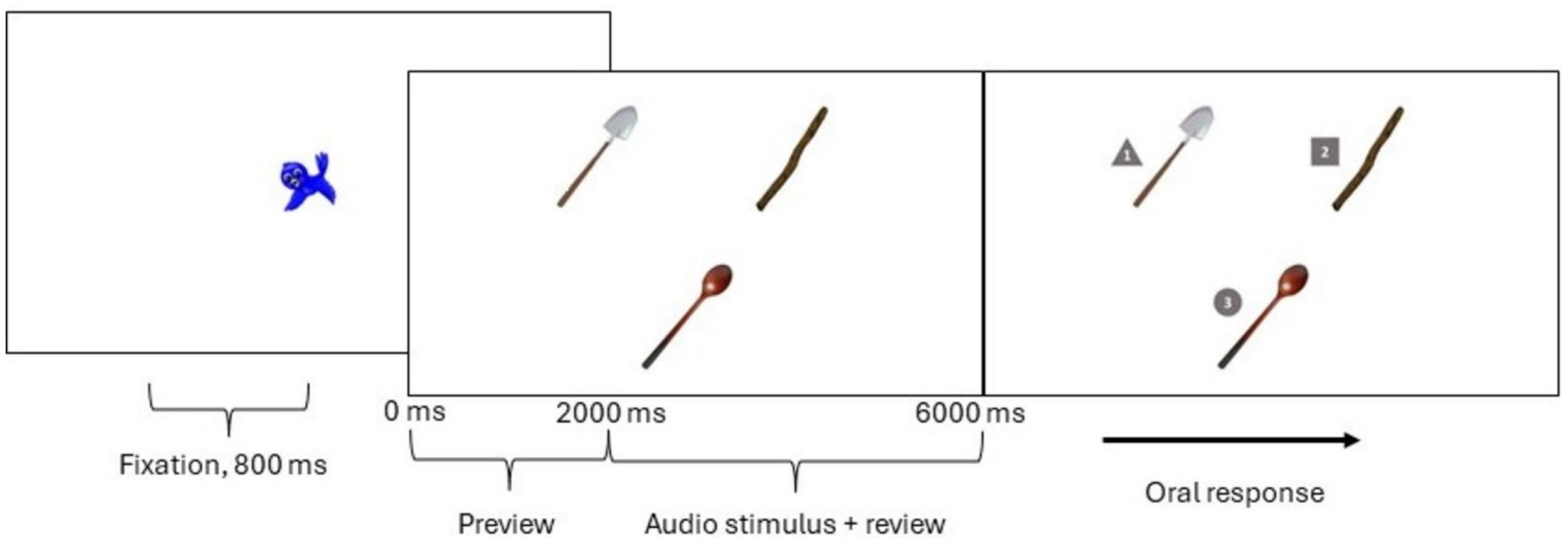
Timeline of eye-tracking procedure.

In piloting, participants appeared more engaged in the task if they were able to answer the question being asked of them, *Where is X object*? Given that we used a 3-object array, we needed to develop a procedure that did not rely on asking participants to click the right/left key of their board or mouse. We also sought to avoid data loss that happens when participants are asked to point with their hands or their cursor, which might block their gaze with their hands, or lead them to looking away. To provide participants with the opportunity to answer (which contributes to data accuracy), and at the same time maintain an uninterrupted gaze at the screen throughout each trial, we asked participants to identify the picture by a number placed adjacent to each image. The numbers 1, 2 and 3 appeared 6 s after image presentation. The location of these numbers was randomized across trials. Once the numbers appeared, the participant could name which number corresponded to the image the bird asked about. Figure 2 illustrates the procedure from fixation to numbered screen for the trial *“¿Dónde está el/mi palo?*” (*Where is the/my stick?*).

The accuracy of the response was manually recorded during the session by the tester, and later verified by a different researcher from the audio recording. Inaccurate trials were excluded from further analysis. Overall, the accuracy rate for participants in this study was 98.8%.

## 3 Results

### 3.1 Analysis

Eye-tracking data was exported in 10 ms time bins using TobiiPro software. Trials with fixations to the three areas of interests were entered for the analysis, and coded as 1 if target, 0 if competitor or distractor. One trial with a transparent, alternating noun was eliminated from analysis because no fixation data was collected for it, due to a technical glitch. The remaining fixation data was fitted to a series of mixed-effects generalized linear mixed models (logit), fit by the maximum likelihood method (Laplace Approximation), using the lme4 package in R (Bates et al., 2015; R Core Team, 2023).

The first three models explored the simple effect of determiner across groups, for each temporal window. These simple models included main effects of group (with ASI as the reference level) and determiner (with possessive as the reference level), the group by determiner interaction, and random effects of participant and item. Given the results of previous studies, we hypothesize a general advantage of the definite determiner, and a general disadvantage in heritage speakers.

The fourth model explored processing of the gender vowel. This model was fitted to a subset of the data where the only expression of gender was the noun-final gender vowel. In other words, this model was restricted to the data on the third temporal window, for trials with transparent (vowel-ending) nouns, accompanied by possessive determiners. We compare the effect of gender in alternating nouns (*palo/pala* ‘stick/shovel’) where the vowel is the only element that differentiates target and competitor, relative to non-alternating nouns (such as singleton *sombrero* ‘hat’, which does not have a minimal pair, and for which the disambiguation for the competitor, *sombrilla* ‘umbrella’, occurs earlier). The variable Gender was added to the model in order to assess whether the specific vowel had an effect (feminine -*a* or masculine -*o*). The model then tested a three-way interaction for group (HSSs vs. ASIs, with ASIs as the reference level), alternation (with non-alternating nouns set as reference level), and gender (with masculine -o set as reference level). As in the previous models, this model included participant and item as random effects. Non-alternating nouns are expected to obtain higher rates of fixations to target, because there is earlier phonological disambiguation from the competitor, which is not a minimal pair. We also anticipate overall group differences (less fixation in the less fluent HSSs). If heritage speakers are less efficient at processing gender or disambiguating vowels, we expect fewer fixations to alternating targets during noun processing, relative to ASIs.

### 3.2 Processing of determiner

The first model tested the effect of determiners across groups during determiner processing (Target ~ Determiner * Group + (1|Participant) + (1|Item)), during Window 1. The model was based on 41,818 observations from 38 participants and 47 items. The model shows a significant intercept (*β* = −0.78, *p* < 0.001), a small but significant main effect of determiner (*β* = 0.14, *p* < 0.001), and no main effect of group (*β* = 0.02, *p* = 0.89). The model also reveals a significant decrease in fixations to target for HSSs in the definite condition (*β* = −0.26, *p* < 0.001). This shows that even at this early phase the definite determiner confers an overall advantage, but HSSs have less looks to target relative to ASIs. To confirm the effect that a given determiner has on target fixations for each group, we used the emmeans package (Lenth, 2024) and conducted post-hoc tests contrasting the estimate marginal means of target fixations for definites vs. possessives for ASIs and HSSs. The results showed that for ASIs, the estimated difference in the marginal means for definites vs. possessives was 0.15 (*z* = 5.14, *p* < 0.01) while for HSSs, the estimated difference was −0.11 (*z* = −3.40, *p* < 0.01). This suggests that ASIs encountering definites had more target fixations compared to possessives, while HSSs encountering definites had fewer target fixations compared to possessives.

Data from the second temporal window, i.e., noun processing, was fitted to a second model, with the same structure as before. The model was based on 68,167 observations from 38 participants over 47 trials. This second model showed a significant intercept (*β* = −0.39, *p* < 0.001), a significant increase of looks to target for the definite (*β* = 0.38, *p* < 0.001), a significant decrease for HSSs (*β* = −0.18, *p* = 0.013), and a significant interaction of group and determiner (*β* = −0.12, *p* < 0.001). During noun processing, participants again looked more to target in definite trials and had overall fewer looks to target if they were heritage speakers. To assess the interaction of determiner and group, we also conducted post-hoc tests using emmeans. For ASIs, the results indicated that the estimated difference in marginal means for definites vs. possessives was 0.38 (*z* = 17.57, *p* < 0.01) while for HSSs, the estimated difference was 0.17 (*z* = 6.87, *p* < 0.01). In other words, both speaker groups experienced more target fixations for definites compared to possessives in Window 2, but the magnitude of the difference between determiners was larger for ASIs than HSSs.

Data from the third temporal window, post speech processing, was entered to a model using the same formula as before. This third model, based on 55,773 observations from 38 participants and 47 trials, indicates a significant intercept (*β* = 0.94, *p* < 0.001), and a significant increase of looks to target for definite determiners (*β* = 0.42, *p* < 0.001). There was no significant effect of group (*β* = −0.28, *p* = 0.17). However, there was a negative interaction: the definite in heritage speakers showed a small but significant decrease in fixations (*β* = −0.08, *p* = 0.047). Posthoc analysis using emmeans showed that for ASIs, the estimated difference in marginal means for definites vs. possessives was 0.42 (*z* = 15.19, *p* < 0.01) while for HSSs, the estimated difference was 0.34 (*z* = 12.13, *p* < 0.01). Just as in Window 2, both speaker groups experienced more target fixations to definites compared to possessives, but the magnitude of the difference between determiners was larger for ASIs than HSSs in the third temporal window.

**Figure 3:**
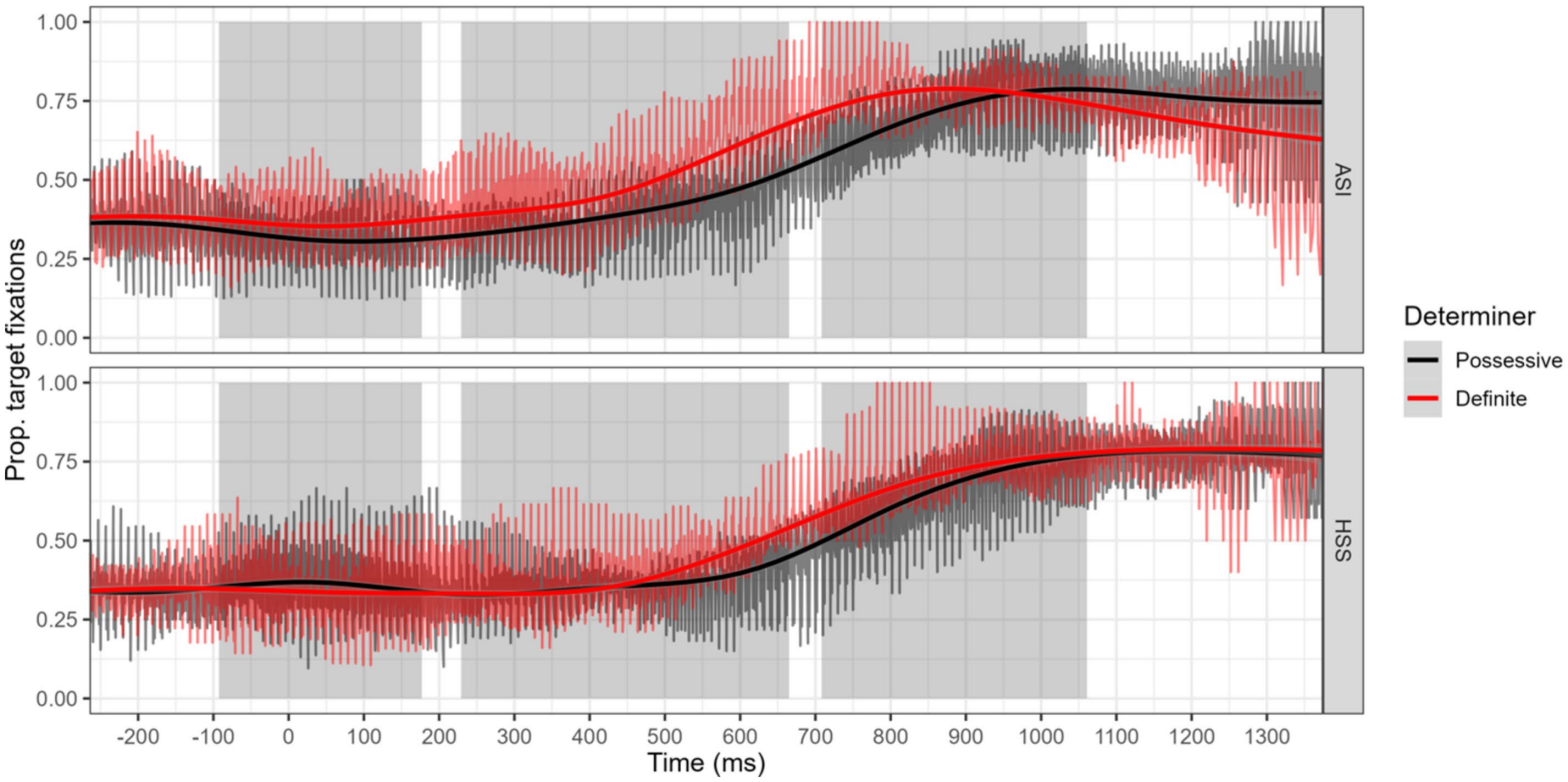
Time course of fixations to target by determiner (possessives and definite) for each group (Adult Spanish Immigrants and Heritage Speakers).

Figure 3 illustrates how at the onset of the first temporal window, both groups of speakers are initially at chance (0.33), but as time progresses fixations to target increase. Crucially, ASIs look more to targets when presented with a definite NP, and this difference tapers during the third window of analysis. In contrast, for heritage speakers the two paths overlap most of the time, with some differentiation becoming apparent towards the end of the second temporal window.

### 3.3 Processing of gender vowel

To examine the processing of the gender vowel we analyzed the subset of data from trials with transparent nouns and possessive prompts, during the third temporal window, that is, after they had just heard the noun-final ending. Data from 17,813 observations, 38 participants and 30 items were fitted to a model with three main effects: group, alternation, and gender. As before, the model contained random effects of participant and item.^5^

This model shows a significant intercept (*β* = 0.88, *p* = 0.007), with no significant main effects for group (*β* = 0.34, *p* = 0.18), alternation (*β* = −0.13, *p* = 0.74), or gender (*β* = 0.37, *p* = 0.34). However, there were significant negative interactions of group and alternation, with heritage speakers fixating less on alternating nouns (*β* = −0.77, *p* < 0.001), and group and gender, with heritage speakers fixating less on feminine nouns (*β* = −0.37, *p* < 0.001). We also found a significant three-way interaction of group, alternation, and gender (*β* = 0.49, *p* < 0.001), but no gender and alternation interaction (*β* = −0.46, *p* = 0.41).

**Figure 4:**
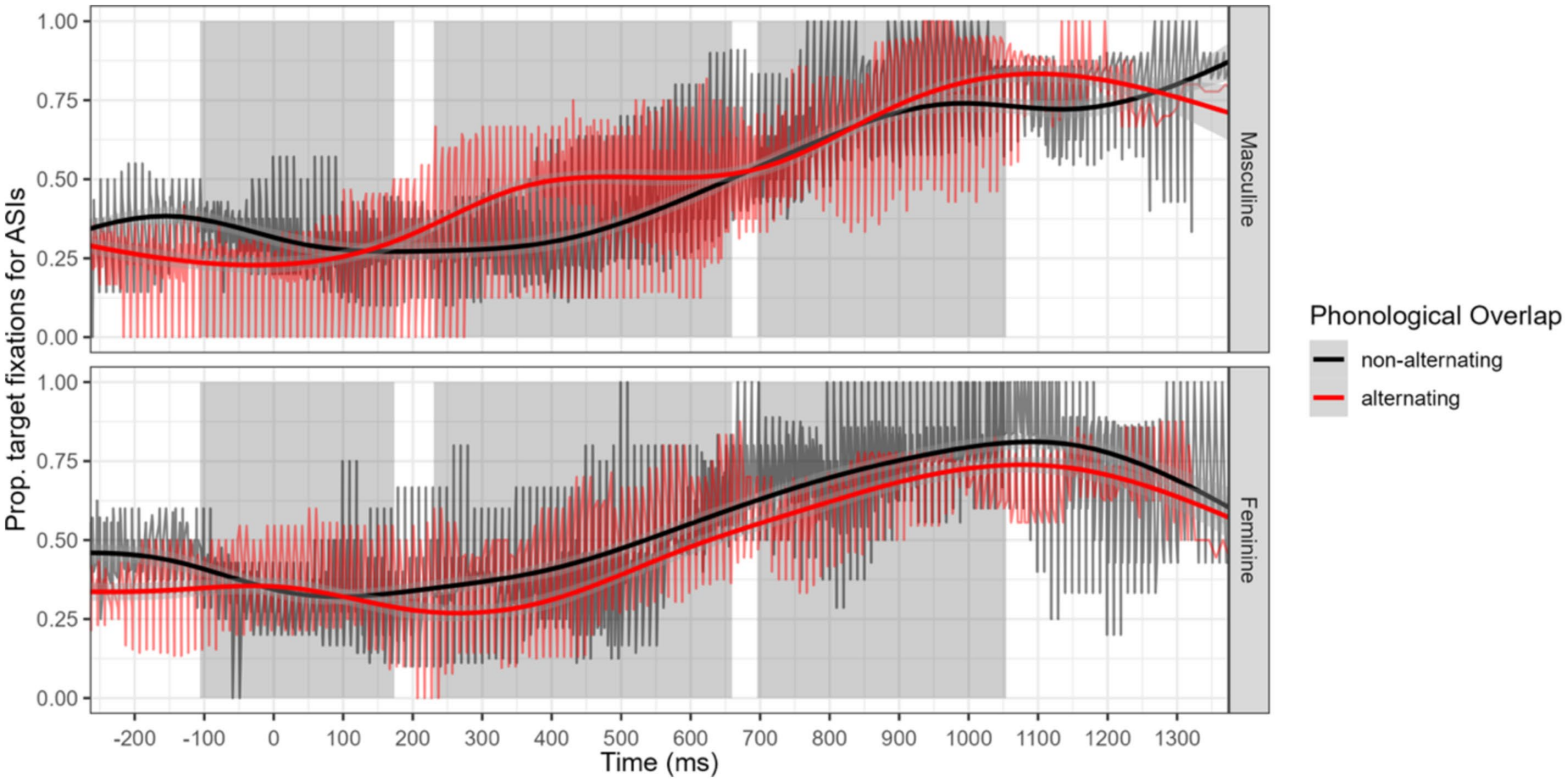
Time course of differences in Adult Spanish Immigrants (ASIs) fixations to target for alternating and non-alternating nouns, represented separately for masculine and feminine gender nouns.

**Figure 5:**
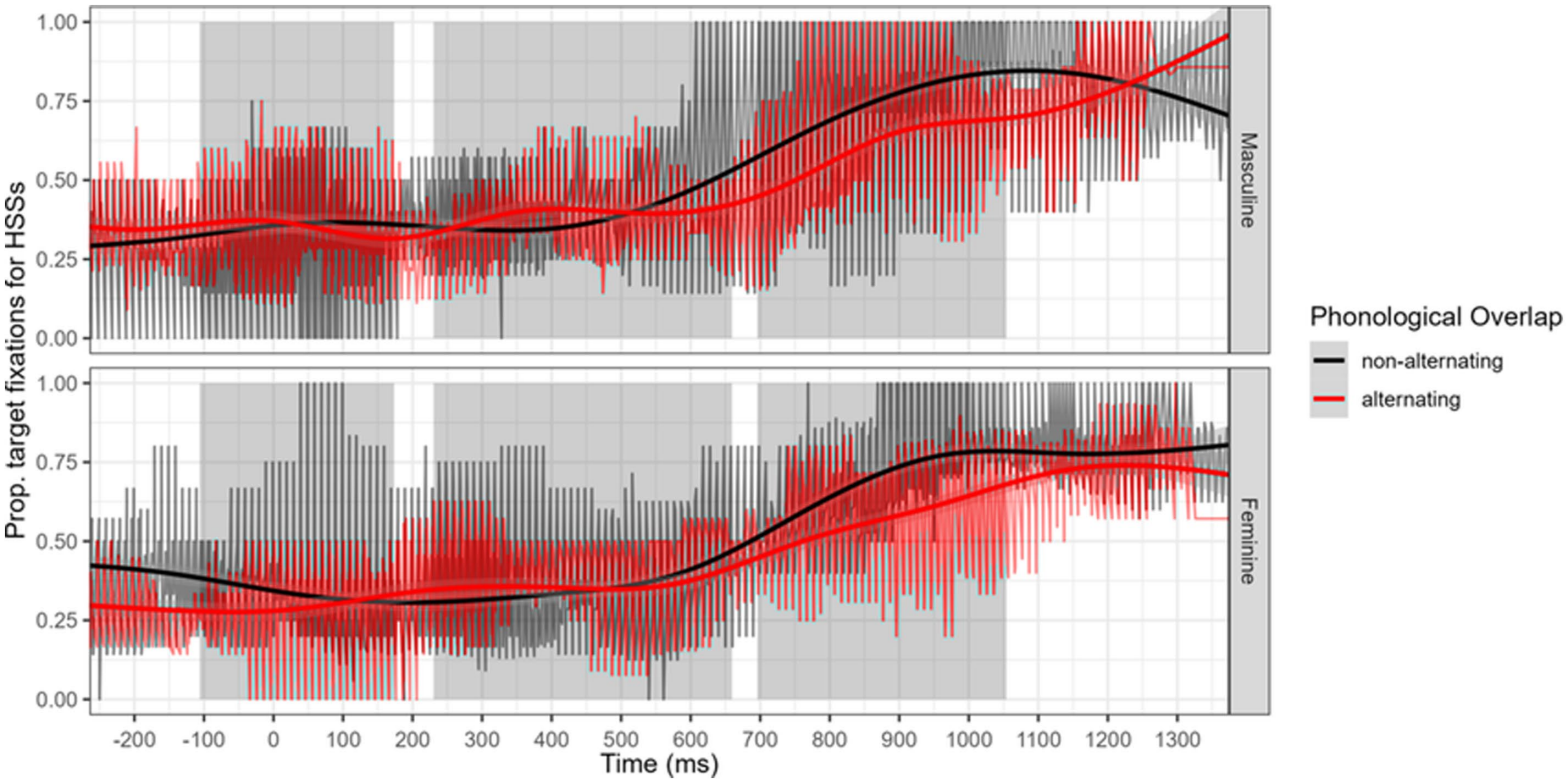
Time course of differences in Heritage Speakers (HSS) fixations to target for alternating and non-alternating nouns, represented separately for masculine and feminine gender nouns.

In examining Figure 4, we note an advantage for non-alternating feminine nouns during post-noun processing, but this advantage did not occur for masculine nouns. Figure 5 indicates that for HSSs, who overall have less fixations than ASIs, both masculine and feminine nouns had higher rates of fixation to non-alternating nouns.^6^

### 3.4 Individual differences

We are particularly interested in examining the heterogeneity within speaker groups. To describe how individuals reacted to the anticipatory advantage of determiner or the processing cost of alternation for gender vowels, we calculated, for each participant, the difference in proportion of fixations to target given the specific linguistic contrast of interest, and then plotted the distribution of participants according to the magnitude of contrast via combined histograms/density plots.

**Figure 6:**
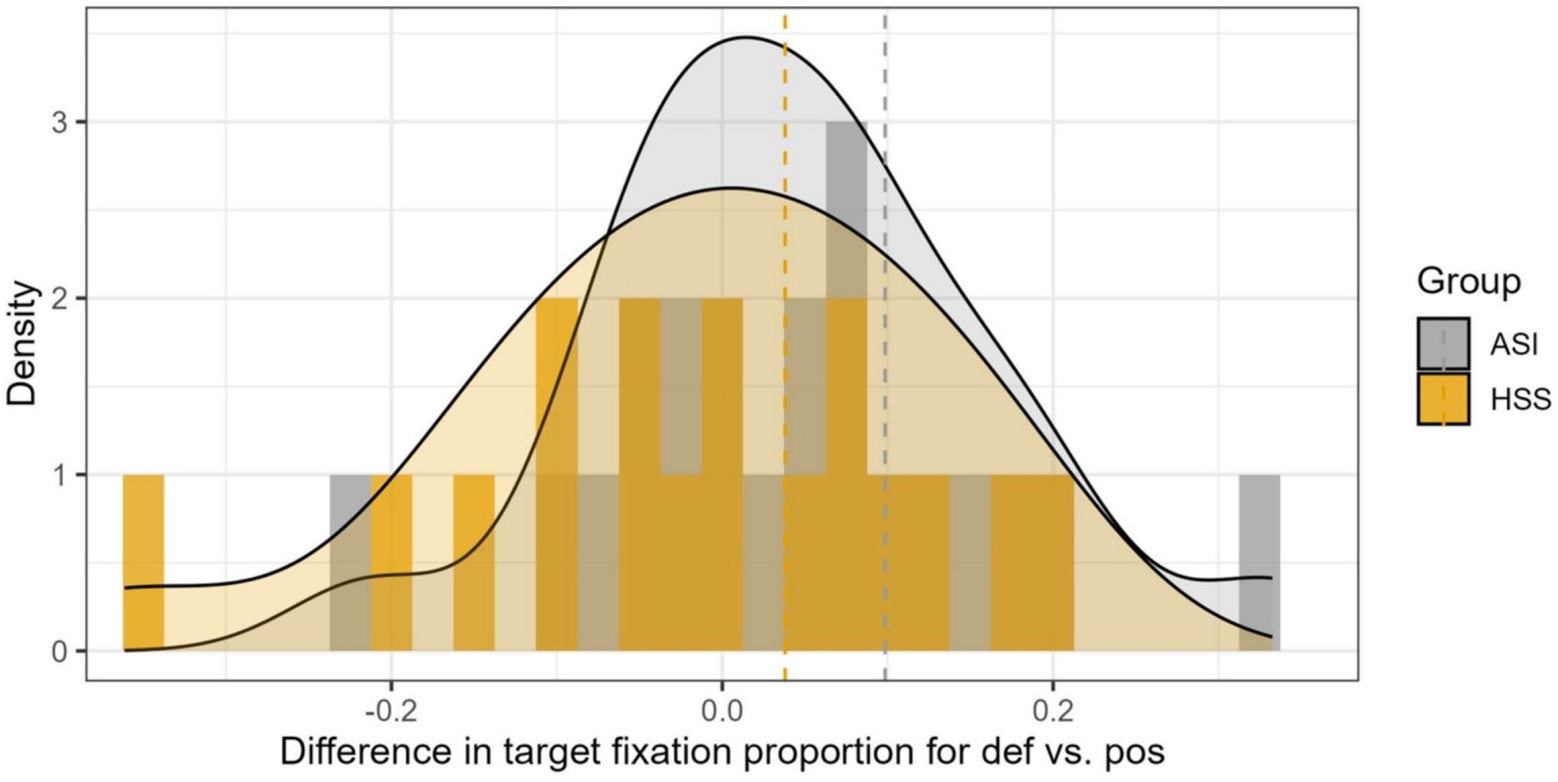
Distribution of heritage (HSS) and adult immigrant speakers (ASI), classified according to magnitude of the difference in fixations to targets in definite and possessive trials for all determiner trials during determiner processing. Dotted line indicates mean differences for each group.

To explore individual differences in the overall anticipatory advantage given by the definite determiner, we subtracted the proportion of fixation to target in definite trials minus possessive trials. For this analysis, we used the proportion of fixation to target during Window 1, as participants were listening to the determiner. Figure 6 illustrates how the two groups are differentiated by the group means and by their outliers: heritage speakers have three of out of the four lowest scores and one adult Spanish immigrant shows the highest advantage score. At the same time, this figure shows substantive overlap in how individuals within the two groups use the determiner.

**Figure 7:**
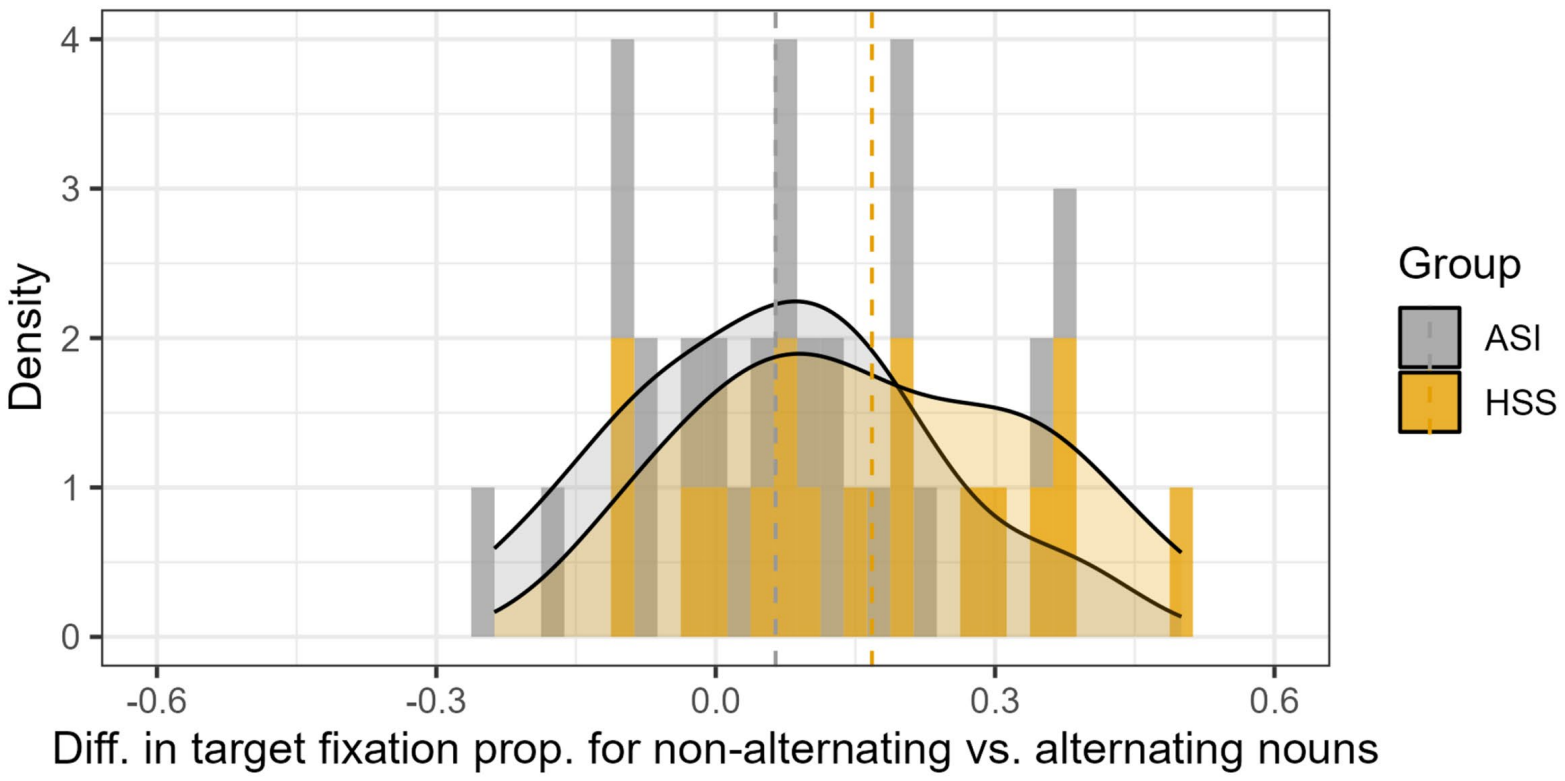
Distribution of heritage and adult immigrant speakers, classified according to the magnitude of the difference in fixations to targets in non-alternating minus alternating trials, for data on gender vowel during post-noun processing. Dotted line indicates mean differences for each group.

To examine individual variation with respect to vowel processing, we calculated the difference scores of the proportion of target fixation during the third temporal window for trials with non-alternating nouns minus that proportion for trials with alternating nouns. We assume that the difference between fixations to non-alternating nouns and alternating nouns represents the difference between lexical retrieval based on word roots, vs. retrieval that requires successful processing of gender vowels. This difference should be larger for bilinguals with less differentiated vocalic space, since such speakers would be less efficient at transparent word endings, where there is closer competition between target and distractor. Lower rates of fixation to alternating nouns will increase the magnitude of the difference in fixations to non-alternating target minus fixations to alternating targets. If this assumption is correct, we expect this analysis to show more HSSs with a larger difference in fixation proportion for different types of nouns. Figure 7 shows that there is a numerical difference in group means, and reveals which individuals have the extreme scores: One HSS has the highest difference scores, and two ASIs have the lowest difference scores. However, as before, most members of the two groups occupy the same range of difference scores.

The purpose of this descriptive data is to illustrate within-group heterogeneity. As expected, for determiner processing, more ASIs take advantage of the determiner. Also as expected, for gender vowel processing, more HSSs speaker show higher differences between non-alternating and alternating nouns. For speakers near or below zero difference scores, we assume that they are not deriving a benefit from the gendered article (for Figure 6), or exhibiting a cost associated with noun alternation. Small, near zero difference scores would reflect unspecified random effects of trial. That a portion of speakers have a positive difference supports the findings detected in the statistical models, but it also shows that under natural speech conditions, many speakers do not manifest the hypothesized contrasts.

## 4 Discussion

The current study first compared the proportion of looks to target nouns presented with definite and possessive determiners, in order to first establish whether Spanish speakers are able to use the gender information on a determiner in naturalistic speech to process an upcoming noun. This allows us to then explore an important question: is this also true of heritage bilinguals, who might be less accurate in gender use, and potentially less adept at using grammatical gender in processing? The results of our exploration on the effect of determiner suggest that even at natural paced speed, speakers are using gender during determiner processing to fixate on the target. This advantage appears early for ASIs, who demonstrate a small but significant effect of the definite during determiner processing that increases during noun processing and tapers off eventually. In contrast, for HSSs, the overall main effect of the definite is moderate. The definite confers no reliable advantage for heritage speakers during determiner processing and differentiation only appears during noun processing, where we observed increased fixations to target in definite trials emerging during the latter part of noun processing. Only in the post speech phase (i.e., Window 3) do we observe a clear difference between determiners, for both groups. To consider the performance of individuals, we generated an individual “advantage score” by subtracting proportion of fixations to target during determiner processing obtained for definite NPs minus proportion of target fixations for possessive NPs. This is intended to reflect the advantage conferred by gender marking in definites in anticipatory looks. When we compare the individual profiles across speaker groups, we note that, while the group mean difference scores are numerically lower for HSSs, many heritage speakers are in a range comparable to those of adult immigrants. We interpret both sets of observations to indicate that some heritage speakers are benefitting from the cue provided by the definite, but overall, as a group, these anticipatory effects are not at the same level as what is observed for ASIs.

Huettig and Guerra (2019) point out that studies examining predictive processing tend to present well-articulated and fairly slow speech to participants. Their study suggests that speech rate matters. Predictive gazes to target in Dutch were only evident when the speech stimuli were presented at a slow speech rate. Participants only exhibited predictive effects for normal speech rate depending on extended preview, and number of objects. We tested the use of gendered determiners in three-object displays and unmanipulated prompts at a normal speech rate with a relative short preview time. Our findings confirm that even with stimuli that are more complex and rely on naturally-paced speech, Spanish bilinguals (including some Heritage Speakers) can take advantage of the information offered by the Spanish definite determiner. At the same time, in both groups, we observe individuals with difference scores at or below zero (Figure 6), implying that not all speakers, even among ASIs, take advantage of the gender marking information.

Our second analysis focused on the processing of the gender vowel in transparent nouns, in contexts where this marker was the only expression of gender. This analysis, performed on the post-speech window, was conducted on the relative difference of fixation to targets when listening to non-alternating nouns (which are phonologically disambiguated earlier) versus alternating nouns (where the gender vowel is the only element that disambiguates target and competitor). There were no overall statistical differences between groups or noun types, but heritage speakers fixated less on alternating nouns, particularly feminine nouns. For heritage speakers, we observed differences in the time-course fixation for both masculine and feminine nouns. In contrast, for adult immigrants, the effect is visible only for feminine nouns. At the individual level, we again observe that most heritage speakers occupy ranges comparable with that of adult immigrants, in terms of the relative disadvantage of alternating nouns when compared to non-alternating nouns. Nonetheless, more heritage speakers have higher difference scores. We interpret this as indication that some HSSs (as well as some ASIs) have less looks to targets when relying only on the word final gender vowels.

The effect of gender deserves further discussion. Heritage speakers showed less fixation to feminine nouns. Our result contributes to ongoing discussions about whether bilinguals and monolinguals represent feminine and masculine nouns differently (see Beatty-Martinez and Dussias, 2019). For bilinguals, gender asymmetries appear to be modulated by previous language knowledge (Dussias et al., 2013) and patterns of Spanish use (Caffarra et al., 2017; Baron et al., 2022). Caffarra et al. (2017) suggest that bilinguals with more “entrenched” grammars (due to greater language use) process grammatical gender more efficiently (i.e., via lexical-based routes), without needing to rely as much on form-based cues, such as transparent morphology. For speakers with less entrenched grammars, masculine and feminine nouns may be processed differently, depending on relative experience and previous language knowledge. However, there are divergent results as to whether masculine or feminine is processed more efficiently, with both types of results accounted for in different types of speakers (Dussias et al., 2013; Fuchs et al., 2021; Baron et al., 2024). An alternative source of interpretation becomes apparent once one considers the phonetic realization of gender vowels. Pérez-Leroux et al. (2023) report that the most common classification error was the misclassified realization of /a/ as /o/. This potentially predicts less perceptual accuracy with feminine /a/. In our study, we identify a processing cost specific to heritage speakers in the contexts where the word-final gender vowel is the only available cue to gender.

Our general goal was to better understand gender processing, and how different types of speakers can quickly integrate visual, acoustic and linguistic information in a more complex experimental setting than the one employed in previous VWP studies. Our results align with previous findings (Lew-Williams and Fernald, 2009; Fuchs, 2022) showing that bilinguals with early exposure to Spanish and advanced communicative abilities as adults can use a gender-marked informative determiner to facilitate noun processing. However, the heritage speakers in our study, whose experience with the Spanish vocalic system includes input with substantive vowel overlap, exhibit a range of performances. Under the more complex conditions in our study (natural speech, and more elaborate visual displays), early bilinguals show an advantage of gendered determiner but only later, during noun processing. Therefore, we cannot rule out that a predictive processing advantage is present for heritage bilinguals. As Huettig (2015, p. 131) suggests, “(p)erhaps prediction is something which happens when cognitive systems have plenty of resources available.” Further analyses are required to understand what conditions systematically provide the ideal context for prediction and processing within the Spanish NP, and how these factors interact with each other based on the language experience of an individual.

Studying language processing and language acquisition in bilinguals offers a unique type of evidence on how variations in human experience affect processes of human language learning and use. We investigated how two different components of gender marking are used by bilinguals while listening to a Spanish noun phrase, including the role of determiners and vocalic word endings, and found both group differences and individual attainment. These findings contribute to a greater understanding of how different sources of information, in this case, grammatical and phonetic, are deployed during sentence comprehension in bilinguals. From an applied perspective, studies that probe bilinguals’ knowledge and use of specific core aspects of grammar expand our understanding of specific areas of difficulty that heritage speakers might encounter with Spanish grammar and literacy skills.

## Data Availability

The raw data supporting the conclusions of this article will be made available by the authors, without undue reservation.

## Footnotes

^1^Valdés Kroff et al. (2017) is a study of code-switching, we refer here to their Spanish-only, non-switched data.

^2^Note that phonetic ambiguity due to co-articulation is restricted to some determiner-noun sequences, such as indefinites or demonstratives followed by a noun starting with a vowel. It does not really arise with definites, where a resyllabified masculine would have an additional syllable compared to a co-articulated feminine (*el amigo/la amiga*).

^3^These two speakers were deemed to have established literacy skills in Spanish. The literature on age effects shows crucial changes in L1 outcomes in immigrants who immigrate during pre-adolescence, and early adolescence. Jia et al.’s (2002) study of Chinese immigrants found better performance on L1 listening was associated with older age of arrival, and onset of English education. In their study, all speakers over age 15 were above 90% accuracy in their Mandarin listening task.

^4^For trials with animal nouns where grammatical gender denotes biological sex: *el perro- la perra* (male dog-female dog), *el oso-la osa* (male bear-female bear), the images had stereotypical “male” or “female” characteristics (e.g., beard, long eyelashes, pink bow). For epicene animal nouns, which have lexically fixed grammatical gender and unspecified biological gender interpretation, the images had no additional visual cues.

^5^We analyzed lexical frequencies to ensure that effects did not arise from frequency asymmetries in noun types (alternating vs. non-alternating targets), or on the contrast between target and competitor. Values were extracted from EsPal for the Latin American Spanish and Subtitle Contextual Diversity corpora (Duchon et al., 2013), using the Zipf Scale (frequency ranking). We found no significant differences between alternating and non-alternating transparent nouns (respective means of 3.68, and 3.74; *t* = −0.295, *df* = 28.1, *p* = 0.769), or for the per-trial contrast between the Zipf score of target minus competitor (alternating = −0.086 and non-alternating = 0.238; *t* = −1.259, df = 27.8, *p* = 0.2185).

^6^As pointed by a Frontiers reviewer, vowel-alternating targets show an advantage in the second window of analysis. This early preference for masculine alternating nouns appears before vowel processing and goes against expectations. While it is not explained by noun target frequency scores masculine alternating nouns are close in frequency to non-alternating noun (3.9 vs. 3.7), a post-hoc exploration shows that for this subset of nouns, the frequency contrast between targets and competitors is higher than what was observed for the whole class of alternating vs. non alternating (0.49), see footnote 6. Note that the same effect can be seen in Figure 5, albeit to a lesser extent.
