# Supplementary material for: The morphophonological dimensions of Spanish gender marking: NP processing in Spanish bilinguals
Source: Front Hum Neurosci. 2024 Dec 13;18:1442339. doi: 10.3389/fnhum.2024.1442339 (PMC11672334; doi:10.3389/fnhum.2024.1442339)
Supplement: Supplementary file 1 [file Data_Sheet_1.pdf]

Appendix 1

**Practice Trials**	
¿Dónde está su pastel?<br>¿Dónde está su gallo?<br>¿Dónde está su papel?<br>¿Dónde está sus tambores?<br>¿Dónde está su oveja?<br>¿Dónde está su mantequilla?	Where is his cake?<br>Where is his rooster?<br>Where is his paper?<br>Where are his drums?<br>Where is his sheep?<br>Where is his butter?)
**Experimental Trials** *(counter-balanced (def/pos), randomized by participant)*	
¿Dónde está la/mi campesina?	Where is the/my farmer (fem)?
¿Dónde está el/mi perro?	Where is the/my dog (masc)?
¿Dónde está la/mi pesa?	Where is the/my weight?
¿Dónde está el/mi palo?	Where is the/my stick?
¿Dónde está la/mi coneja?	Where is the/my rabbit (fem)?
¿Dónde está el/mi brujo?	Where is the/my witch (masc)?
¿Dónde está el/mi ramo?	Where is the/my bouquet?
¿Dónde está la/mi puerta?	Where is the/my door?
¿Dónde está la/mi cocinera?	Where is the/my cook (fem)?
¿Dónde está el/mi oso?	Where is the/my bear (masc)?
¿Dónde está el/mi ojo?	Where is the/my eye?
¿Dónde está la/mi raya?	Where is the/my line?
¿Dónde está la gata?	Where is the/my cat (fem)?
¿Dónde está el/mi maestro?	Where is the/my teacher (masc)?
¿Dónde está la/mi vela?	Where is the/my candle?
¿Dónde está el/mi plato?	Where is the/my plate?
¿Dónde está la/mi princesa?	Where is the/my princess?
¿Dónde está el/mi toro?	Where is the/my bull?
¿Dónde está la/mi libreta?	Where is the/my notebook?
¿Dónde está el/mi coco?	Where is the/my coconut?
¿Dónde está la/mi niñera?	Where is the/my nanny?
¿Dónde está el/mi camello?	Where is the/my camel?
¿Dónde está la/mi caja?	Where is the/my box?
¿Dónde está el/mi jabón?	Where is the/my soap?
¿Dónde está la/mi ballena?	Where is the/my whale?
¿Dónde está el/mi caballero?	Where is the/my knight?
¿Dónde está la/mi piña?	Where is the/my pineapple?
¿Dónde está el/mi zapato?	Where is the/my shoe?
¿Dónde está la/mi mosca?	Where is the/my fly?
¿Dónde está el/mi sapo?	Where is the/my toad?
¿Dónde está la/mi casa?	Where is the/my house?
¿Dónde está el/mi sombrero?	Where is the/my hat?
¿Dónde está la/mi cantante?	Where is the/my singer (fem)?
¿Dónde está el/mi león?	Where is the/my lion?
¿Dónde está la/mi nube?	Where is the/my cloud?
¿Dónde está el/mi cofre?	Where is the/my treasure box?
¿Dónde está la/mi serpiente?	Where is the/my snake?
¿Dónde está el/mi paciente?	Where is the/my patient (masc)?

¿Dónde está la/mi leche?	Where is the/my milk?
¿Dónde está el/mi sobre?	Where is the/my envelope?
¿Dónde está la/mi mujer?	Where is the/my woman?
¿Dónde está el/mi escorpión?	Where is the/my scorpion?
¿Dónde está la/mi sal?	Where is the/my salt?
¿Dónde está el/mi tren?	Where is the/my train?
¿Dónde está la/mi lombriz?	Where is the/my worm?
¿Dónde está el/mi doctor?	Where is the/my doctor (masc)?
¿Dónde está la/mi red?	Where is the/my net?
¿Dónde está el/mi camión?	Where is the/my truck?
